# Late Relapse of Henoch-Schönlein Purpura in an Adolescent Presenting as Severe Gastroduodenitis

**DOI:** 10.3389/fped.2018.00355

**Published:** 2018-11-20

**Authors:** Chiara Rubino, Monica Paci, Massimo Resti, Paolo Lionetti, Sandra Trapani

**Affiliations:** ^1^Pediatric Department, Anna Meyer Children's Hospital, Florence, Italy; ^2^Gastroenterology Unit, Anna Meyer Children's Hospital, Florence, Italy

**Keywords:** Henoch-Schönlein purpura, duodeno-jejunitis, relapse, atypical presentation, endoscopy

## Abstract

Henoch-Schönlein purpura is a systemic vasculitis, commonly affecting children. Gastrointestinal manifestations are observed in 50–75% of patients; it is well known they may occur before skin lesions in about 20% of cases during the first vasculitic episode. Relapses occur in about one third of patients, typically within 4 months from the initial presentation and with milder symptoms. We report the case of a 17-year old girl with an atypical relapse of Henoch-Schönlein purpura, presenting with acute abdominal symptoms 5 years after the first episode. Esophagogastroduodenoscopy showed duodenal multiple hyperemic and hemorrhagic lesions. To our knowledge this is the first case of hemorrhagic-erosive duodenitis representing a relapse of Henoch-Schönlein purpura occurring several years after the initial episode. Duodenojejunal inflammation should be considered as primary manifestation of Henoch-Schönlein purpura, not only during the first episode, but also in relapses. Endoscopy can be helpful for differential diagnosis, especially in patients with atypical manifestations. Further studies are needed to evaluate risk factors for Henoch-Schönlein purpura recurrence and the possible role of fecal calprotectin as an early marker for gastrointestinal involvement.

## Background

Henoch-Schönlein purpura (HSP) is a systemic small vessel vasculitis commonly affecting children. Gastrointestinal (GI) manifestations are frequent and they generally involve the small intestine. Duodeno-jejunitis can be the main clinical manifestation of HSP and in about 20% of cases it may appear before skin lesions during the first episode (1). It may be difficult to diagnose HSP based merely on GI symptoms if palpable purpura is absent. Relapses of HSP are common and they usually present as a new flare of cutaneous lesions, followed by gastrointestinal or renal impairment.

The present report describes an unusual late onset relapse of HSP with gastroduodenitis diagnosed with endoscopy.

## Case presentation

### Presenting concerns

A 17-year old girl was admitted to a local hospital for acute abdominal symptoms. She had a previous history of HSP with typical cutaneous and articular manifestations and good response to oral steroid therapy, at the age of 12. Ten months after the first HSP occurrence, she relapsed presenting the same clinical features. Renal involvement was always absent. At age 16 she developed erythema nodosum which was successfully treated with oral steroid therapy.

On admission to the local hospital she presented with fever, vomiting, diarrhea and epigastric pain. Symptoms had begun 5 days before and had gradually worsened. She was not under any medications and did not report any recent infection. Physical examination was unremarkable except for abdominal pain on deep palpation especially in the epigastric region, without hepatomegaly or splenomegaly; Blumberg and Murphy signs were negative. There was no skin rash at admission.

Laboratory analysis showed slightly increased C-reactive protein (CRP) (2.3 mg/dL, normal value < 0.29 mg/dl). During hospitalization, gastrointestinal symptoms worsened despite intravenous (IV) proton pump inhibitor and empiric antibiotic treatment. Three days after admission, a few petechiae appeared on arms and feet. The patient was thus transferred to our hospital.

### Clinical findings

On arrival she was febrile and presented continuous projectile vomiting. Vital parameters were normal except for mild tachycardia (140 bpm). Few petechial lesions were present on forearms, hands, and insteps. Lungs and heart examination was unremarkable. The abdomen was diffusely painful and tender, especially in upper quadrants, without rebound tenderness or organomegaly. Rectal examination did not show any bleeding, anal fissures, ulcers, abscesses, fistulae or scarring. Musculoskeletal examination was also normal.

### Diagnostic focus and assessment

Blood exams were unremarkable, except for mild leukocytosis (leucocytes 18,120/mm^3^) and elevated CRP (4.36 mg/dL). Erythrocyte sedimentation rate (ESR), procalcitonin (PCT), transaminases, total and direct bilirubin, amylase, lipase, coagulation profile were within normal range. Stool culture, testing for Rotavirus and Adenovirus, *Clostridium difficilis* toxin and antigen were negative. Hemoccult was positive on three stools specimens. Urinalysis showed no significant abnormalities. Anti-*Saccharomyces cerevisiae* antibodies (ASCA) and anti-neutrophil cytoplasmic antibodies (ANCA), were performed in the workout for inflammatory bowel diseases and resulted negative. On the other hand, fecal calprotectin dosage was elevated (>300 mg/Kg, normal value: <50 mg/Kg). Immunological laboratory tests (IgG, IgA, IgM levels, IgG subclasses, and lymphocyte subpopulations) were normal.

Abdomen ultrasound showed scarce peristalsis, slight ascites, hypervascularization and wall thickening of pylorus and second portion of duodenum. Plain abdomen X-ray and CT scan showed no significant abnormalities.

**Figure 1:**
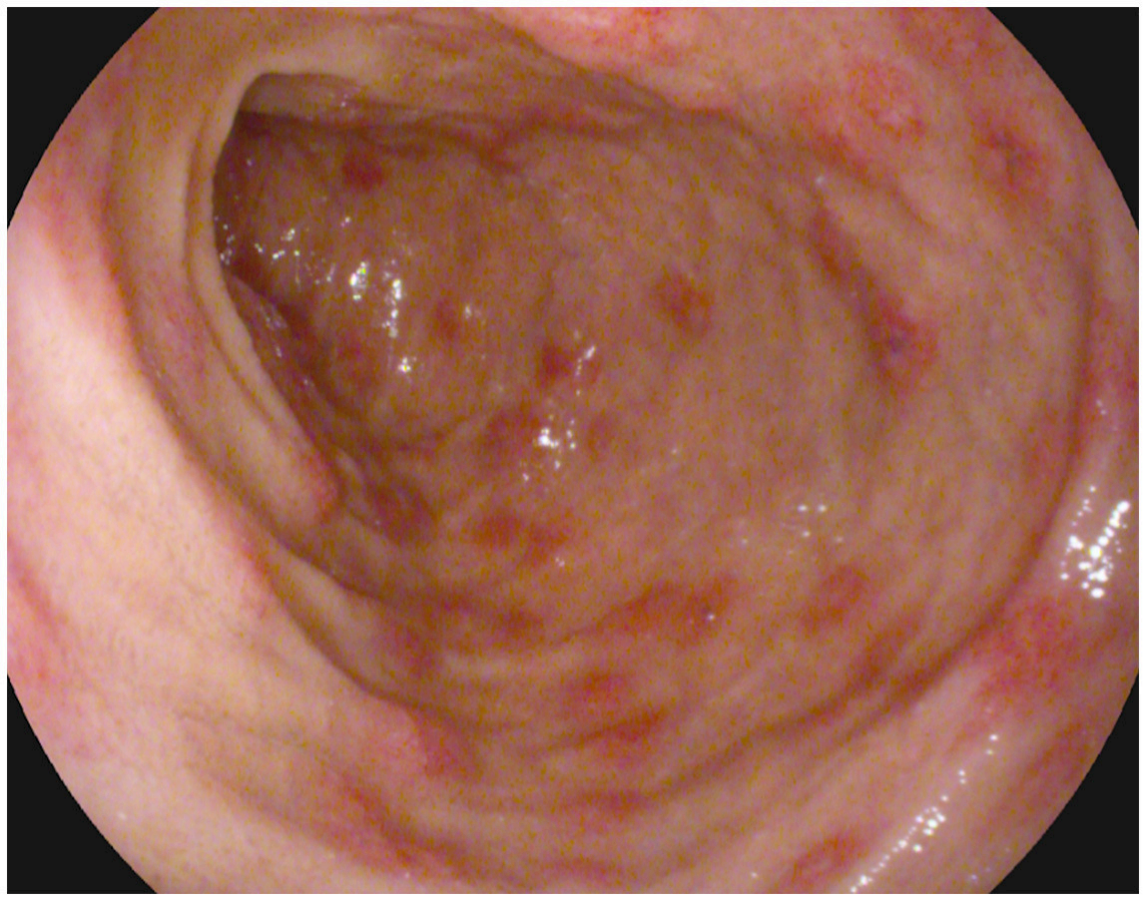
Esophagogastroduodenoscopy evidence of multiple hyperemic and hemorrhagic round-shaped lesions in distal duodenum.

**Figure 2:**
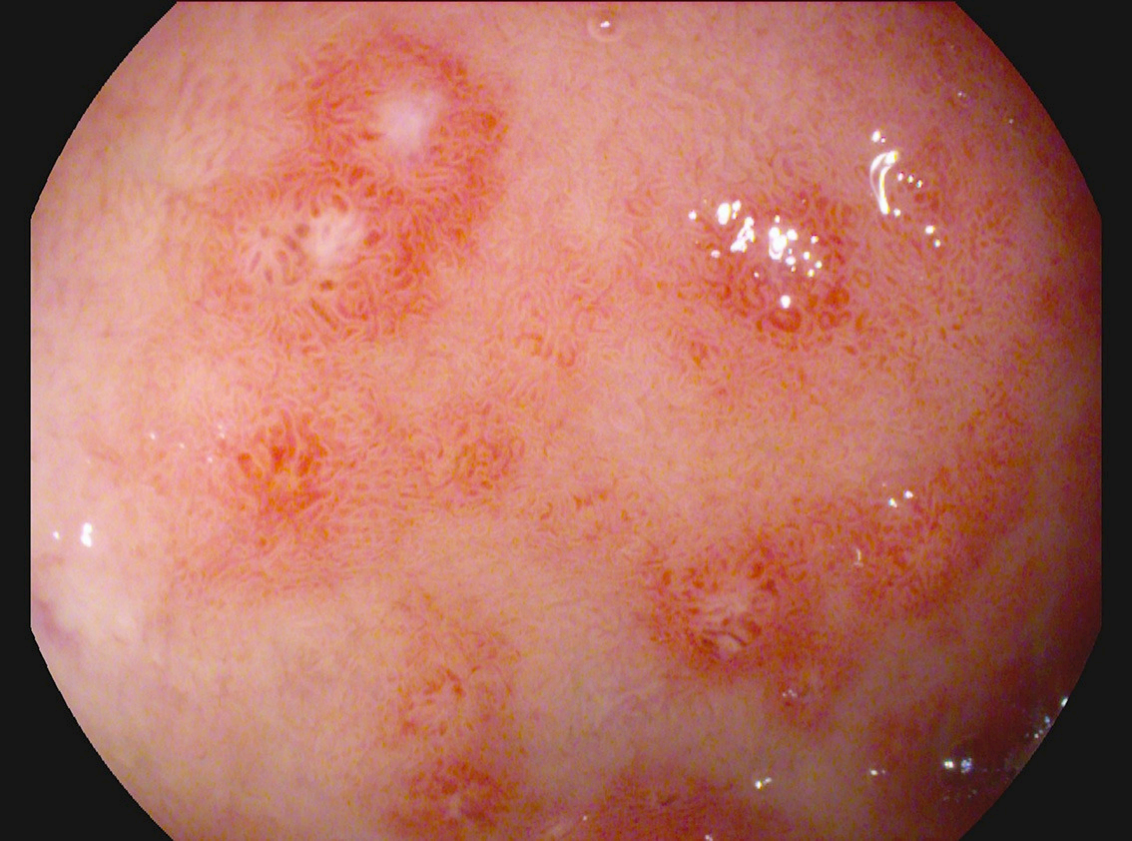
Detail of duodenal lesions (PENTAX i-scan imaging).

**Figure 3:**
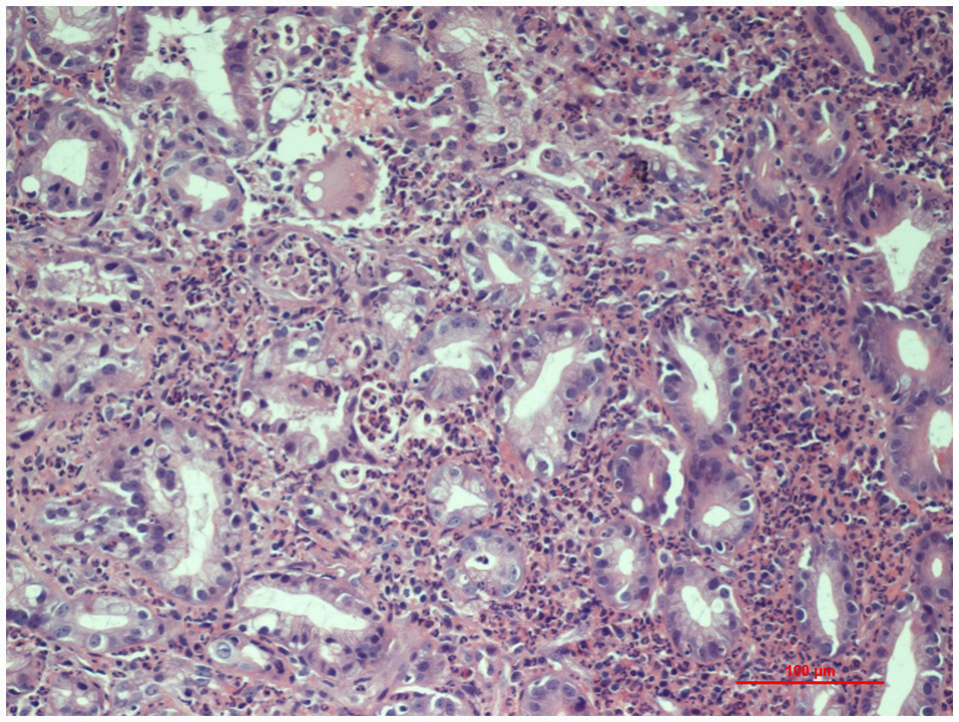
Histopathology of gastric mucosa showing active inflammation (Hematoxylin and Eosin staining, 20x magnification).

For the persistence of abdominal pain, an esophagogastroduodenoscopy was performed, which revealed pyloric edema, multiple hyperemic and hemorrhagic lesions with round shape in the duodenal bulb and descending duodenum, some of them were ulcerating (Figures 1, 2). Histological examination showed active but non-specific inflammation with eosinophilic component and IgA deposition (Figure 3).

Overall, clinical manifestations, the results of laboratory analysis and the macroscopic and histopathological aspects of pylorus and duodenum, prompted the diagnosis of HSP relapse with initial GI involvement.

### Therapeutic focus and assessment

On admission, the patient was started on total parenteral nutrition and IV antibiotic treatment with ceftazidime (150 mg/kg/day IV in three divided doses) and metronidazole (40 mg/kg/day IV in three divided doses).

After endoscopy, high-dose IV methylprednisolone (30 mg/Kg/day) for 3 days, followed by oral steroids (prednisone 1 mg/kg/day) was started with dramatic improvement of gastrointestinal and cutaneous symptoms, which disappeared within a week. Enteral nutrition was gradually started again and she was discharged on oral prednisone.

### Follow-up and outcome

Four weeks later, abdomen ultrasound did not show any abnormalities. Periodic urinalysis and fecal occult blood tests were normal. Oral steroid therapy was gradually tapered in 6 weeks.

At 1 year follow up, the patient was asymptomatic and showed no recurrence of skin rash or gastrointestinal symptoms.

Written informed consent was obtained from the patient's parents for the publication of this case report. As the patient's age was 17 years, we also obtained her informed consent.

## Discussion

HSP is the most common systemic vasculitis in childhood. The mean age of presentation is 6 years with most cases in children under 10 years of age (2).

Diagnosis of HSP is made in a patient with palpable purpura (mandatory criterion) not related to thrombocytopenia, with lower limb predominance, in the presence of at least one of the following: diffuse abdominal pain; any biopsy showing typically leucocytoclastic vasculitis with predominant IgA deposit or proliferative glomerulonephritis with predominant IgA deposit; arthritis or arthralgia; renal involvement (hematuria and/or proteinuria). For purpura with atypical distribution a demonstration of an IgA deposition through biopsy is required (3).

HSP is a leukocytoclastic vasculitis accompanied by IgA immune complexes within affected organs; the predominant cell types in the inflammatory infiltrate are neutrophils (4). Its pathogenesis is still unclear but it is considered a complex disease contributed by several genetic and environmental factors. No confirmed genetic loci have been found, although some studies have described an association with some HLA polymorphisms, cytokines and adhesion molecules, renin-angiotensin system, ß-1,3-galactosyltransferase (5). IgA, the main component of the immune deposits in HSP, can activate the complement through the mannan-binding lectin and alternative pathways and it can bind and activate several receptors (transferrin receptor, soluble receptor and transmembrane receptor of IgA), implicated in HSP pathogenesis (6). Recently, an aberrant glycosylation of IgA1 (the most represented IgA subclasses) has been proposed has an important mechanism in HSP pathogenesis and IgA nephropathy. As regards triggers of HSP, the majority of HSP pediatric cases follow a bacterial or viral infection and upper respiratory tract infections are present in 35–52% of patients. An association between HSP and vaccines, drugs and malignancies has also been reported, the last two especially in adults (4). No recent infection, medication use has been reported in our case and immunodeficiency/immunodisregulation diseases have been ruled out by normal immunological workout.

Gastrointestinal symptoms occur in 50–75% of children with HSP (1). These manifestations result from submucosal hemorrhages and edema of the bowel wall caused by the underlying vasculitis (2). The small intestine is the most frequently involved site in the gastrointestinal tract. The second portion of duodenum is involved more frequently than the bulb, with duodeno-jejunal inflammation as predominant feature (4). Abdominal pain is the most common symptom in HSP and occurs in 60–65% of patients (7). Pain is typically colicky and localized to periumbilical and epigastric regions and worsens after meals, mimicking bowel angina or ischemia. The abdomen may be tender or resemble an acute abdomen, often resulting in unnecessary surgery. Vomiting and GI bleeding (gross or occult blood per rectum) occurs in about one third of pediatric patients (1, 4, 7).

Rarely, (ileo-ileal) intussusception, bowel wall ischemic necrosis, intestinal perforation, acute acalculous cholecystitis, hemorrhagic ascites with serositis, pancreatitis, and biliary cirrhosis may occur (4, 7).

Endoscopic, radiological, and ultrasound studies are used in the diagnosis of GI complications in HSP. Abdomen ultrasound is considered the initial test and reveals bowel wall thickening, reduced peristalsis and dilated bowel loops; moreover, it can rule out surgical complications such as intussusception or perforation. Second-line radiologic exams include CT scan and MRI (8).

As for our case, endoscopy can provide definitive diagnosis, especially when abdominal symptoms precede cutaneous lesions (9). Endoscopic findings include redness, swelling, petechiae, submucosal hemorrhage, purpura, erosions, and ulcerations of the mucosa. Several studies demonstrate the role of endoscopy in GI presentation of vasculitis. Gong et al. (10) demonstrated the presence of erosion, petechiae, submucosal hemorrhage, or ulcers in 95.9% of the patients diagnosed as primary vasculitis with upper GI involvement, and the second portion of the duodenum was the most frequently involved site. The authors conclude that combining endoscopic findings with clinical and radiological features can facilitate diagnosis of vasculitis with upper GI tract involvement. Kishikawa et al. (11) described two adult patients presenting with acute GI presentation, whose endoscopic exams (colonscopy, capsule endoscopy, single balloon enteroscopy) showed circular reddish lesions, some of which ulcerating, and allowed diagnosis of HSP. The authors suggest that these lesions are a characteristic of HSP in the gastrointestinal tract, including the small intestine.

Intestinal biopsies show IgA depositions in 64% of patients and vasculitis may be found in submucosal intestinal vessels (4). Histological findings range from non-specific inflammation to ulceration, edema, hemorrhage, and vascular congestion (4). Several studies show that endoscopic biopsy has low sensitivity to diagnose vasculitis because most biopsies are limited to superficial mucosa and do not reach deeper vessels. However, mucosal biopsies can be essential to exclude other diseases, such as inflammatory bowel diseases. In the study by Gong et al. (10), only 5.4% of the 124 biopsies taken from upper GI tract showed evidence of vasculitis. Zhang et al. (12) analyzed endoscopic biopsies from 54 patients with HSP and gastrointestinal symptoms: all the biopsies demonstrated non-specific inflammation. Our patient's biopsy did not show leukocytoclastic vasculitis but it showed IgA deposition. It also revealed active inflammation with eosinophilic component. The presence of an eosinophilic infiltrate in HSP duodenitis was also described by Louie et al. in three of the 16 duodenal biopsies of HSP patients (13).

In the presence of severe GI symptoms, infectious gastroenteritis, eosinophilic gastroenteritis, Crohn's disease, ulcerative colitis, microscopic polyangiitis, Wegener's granulomatosis and immunodeficiency should be considered in the differential diagnosis (14). Immunodeficiencies should be considered in cases of frequently relapsing gastrointestinal symptoms, especially in early onset cases and in association with frequent infections and hematological alterations. Distinguishing HSP with GI onset from Crohn's disease can be challenging. In our case, the age of the patient, the history of erythema nodosum, the acute GI presentation and the elevation of fecal calprotectin could suggest the onset of Crohn's disease. On the other side, the previous episode of HSP, major gastroduodenal involvement and subsequent appearance of petechiae oriented us toward GI HSP. In these cases, endoscopy is essential to the differential diagnosis. Compared to HSP, Crohn's disease rarely affects duodenum and jejunum, more likely results in fibrosis and stricture formation, usually does not manifest as edema and intramural hemorrhage and does not have IgA deposition (4).

Similarly to our case, recent studies demonstrate that fecal calprotectin can be elevated in acute phases of gastrointestinal involvement in HSP. Therefore, it has been proposed as a marker for early diagnosis of GI involvement in HSP (15, 16).

It is well known that gastrointestinal symptoms may precede the purpura by up to 2 weeks in about 20% of children presenting a first HSP episode (1, 14), with the cutaneous rash commonly occurring about 1 week (average 6.6 days) after the onset of abdominal pain (17). However, no data are available about gastrointestinal clinical presentation, without cutaneous lesions, during relapses.

In most patients, HSP has an excellent prognosis with spontaneous resolution of symptoms. Relapses occur in about one third of patients, after an interval of 4 months to 1 year from the initial presentation (18). Lei et al. (18) defined relapse as a second HSP diagnosis at least 3 months apart from the first episode: the definition of this interval was based on findings from several epidemiological studies. However, according to previous studies (8, 19), the time elapsed between onset and relapse is defined as 1 month. Clinical features at the time of the relapses resemble the ones at disease diagnosis and in most cases subsequent episodes are milder and shorter (2, 19).

Risk factors influencing relapses are not definitely established due to the extreme variability in different studies. Lei et al. found that renal involvement, underlying allergic rhinitis and steroid treatment lasting for >10 days are risk factors for HSP recurrence in children (18). However, results about long-term effects of steroid use in HSP are discordant and the relationship between steroid use and recurrence has not been established (19–21). Therefore, these drugs should be used with caution in HSP. In the hospital setting, early corticosteroid exposure is associated with benefits for several clinically relevant HSP outcomes, specifically those related to the gastrointestinal manifestations (22). Thus, as in our case, severe GI manifestations are a common indication for steroid treatment.

We present the case of a 17-year-old girl transferred to our hospital for persistent vomiting and epigastric pain. Surgical emergencies were excluded. As to differentiate between abdominal HSP relapse and Crohn's disease an esophagogastroduodenoscopy with biopsy was performed and results were suggestive of HSP.

There are previous reports in literature describing presentation of HSP with duodenitis in children and adults, whose diagnosis was possible through a high index of suspicion in front of acute GI manifestations and the findings of esophagogastroduodenoscopy (7, 14, 23–26).

In our opinion, the case we presented adds several points of interest compared to previously published cases. First of all, the patient's age and the 5-year period between the first episode of HSP and the relapse are unusual. In addition, clinical manifestations of the relapse do not resemble the features of the initial episode and are more severe.

In the literature there is only another case report describing a patient presenting hemorrhagic-erosive duodenitis as the first sign of HSP relapse (27). However, the patient had a typical age for HSP (7 years old), a typical interval between disease onset and relapse (13 months), and the same clinical features in the 2 episodes. Therefore, if compared to the cited case, our patient still presents several atypical aspects.

Thus, duodeno-jejunal inflammation should be considered as primary manifestation of HSP, not only during the initial episode, but also in relapses. Endoscopy can support the diagnosis of gastrointestinal involvement in HSP, especially in patients without typical skin rash, either in the first or in the relapsing episodes of the disease.

Further studies are needed to evaluate risk factors for HSP recurrence and the possible role of fecal calprotectin as an early marker for GI involvement.

## Concluding remarks

The diagnosis of HSP can be challenging, especially when the abdominal symptoms precede the characteristic palpable purpura and in atypical clinical scenarios. HSP should be considered in differential diagnosis for patients with abdominal pain and intestinal bleeding, even in the absence of initial cutaneous involvement. Typical endoscopic findings may alert gastroenterologists to consider this disease.

## Author contributions

CR and ST gave a substantial contribution in article conception and design. MP participated in acquisition of data. CR and ST drafted the manuscript; MP, PL, and MR critically revised it. All the authors gave their final approval to this manuscript and agree to be accountable for all aspects of work ensuring integrity and accuracy.

### Conflict of interest statement

The authors declare that the research was conducted in the absence of any commercial or financial relationships that could be construed as a potential conflict of interest.

## References

[1] Weiss PF. Pediatric vasculitis. Pediatr Clin North Am. (2012) 59:407–3. 10.1016/j.pcl.2012.03.013 22560577 PMC3348547

[2] Trnka P. Henoch–Schönlein purpura in children. J Paediatr Child Health (2013) 49:995–1003. 10.1111/jpc.12403 24134307

[3] Ozen S Pistorio A Iusan SM Bakkaloglu A Herlin T Brik R. EULAR/PRINTO/PRES criteria for Henoch-Schonlein purpura, childhood polyarteritis nodosa, childhood Wegener granulomatosis and childhood Takayasu arteritis: Ankara 2008. Part II: final classification criteria. Ann Rheum Dis. (2010) 69:798–806. 10.1136/ard.2009.116657 20413568

[4] Ebert EC. Gastrointestinal manifestations of Henoch-Schonlein Purpura. Dig Dis Sci. (2008) 53:2011–9. 10.1007/s10620-007-0147-0 18351468

[5] Park SJ Suh JS Lee JH Lee JW Kim SH Han KH. Advances in our understanding of the pathogenesis of Henoch-Schönlein purpura and the implications for improving its diagnosis. Expert Rev Clin Immunol. (2013) 9:1223–38. 10.1586/1744666X.2013.850028 24215411

[6] Heineke MH Ballering AV Jamin A Ben Mkaddem S Monteiro RC Van Egmond M. New insights in the pathogenesis of immunoglobulin A vasculitis (Henoch-Schönlein purpura). Autoimmunity Rev. (2017) 16:1246–53. 10.1016/j.autrev.2017.10.009 29037908

[7] Ofori E Ramai D Ona MA Papafragkakis C Reddy M. Adult-onset henoch-schonlein purpura duodenitis. J Clin Med Res. (2017) 9:958–61. 10.14740/jocmr3181w 29038676 PMC5633099

[8] Lee YL Kim YB Koo JW Chung JY. Henoch-Schonlein purpura in children hospitalized at a tertiary hospital during 2004-2015 in Korea: epidemiology and clinical management. Pediatr Gastroenterol Hepatol Nutr. (2016) 19:175–85. 10.5223/pghn.2016.19.3.175 27738599 PMC5061659

[9] Esaki M Matsumoto T Nakamura S Kawasaki M Iwai K Hirakawa K. GI involvement in Henoch-Schonlein purpura. Gastrointest Endosc. (2002) 56:920–3. 10.1016/S0016-5107(02)70376-3 12447314

[10] Gong EJ Kim do H Chun JH Ahn JY Choi KS Jung KW. Endoscopic findings of upper gastrointestinal involvement in primary vasculitis. Gut Liver (2016) 10:542–8. 10.5009/gnl15198 27226428 PMC4933413

[11] Kishikawa H Nishida J Takarabe S Arahata K Ito A Miyoshi J “Circular reddish lesions”: a possibly characteristic endoscopic finding in Henoch-Schönlein purpura. Endoscopy (2013) 45(Suppl 2) UCTN:E33–4. 10.1055/s-0032-1325886 23526504

[12] Zhang Y Huang X. Gastrointestinal involvement in Henoch-Schonlein Purpura. Scand J Gastroenterol. (2008) 43:1038–43. 10.1080/00365520802101861 18609159

[13] Louie CY Gomez AJ Sibley RK Bass D Longacre TA. Histologic features of gastrointestinal tract biopsies in iga vasculitis (Henoch-Schönlein Purpura). Am J Surg Pathol. (2018) 42:529–33. 10.1097/PAS.0000000000001036 29438165

[14] Kang HS Chung HS Kang KS Han KH. High-dose methylprednisolone pulse therapy for treatment of refractory intestinal involvement caused by Henoch–Schönlein purpura: a case report. J Med Case Rep. (2015) 9:65. 10.1186/s13256-015-0545-4 25885905 PMC4378549

[15] Kanik A Baran M Ince FD Cebeci O Bozkurt M Cavusoglu D. Faecal calprotectin levels in children with Henoch-Schönlein purpura: is this a new marker for gastrointestinal involvement? Eur J Gastroenterol Hepatol. (2015) 27:254–8. 10.1097/MEG.0000000000000284 25629568

[16] Teng X Gao C Sun M Wu J. Clinical significance of fecal calprotectin for the early diagnosis of abdominal type of Henoch-Schonlein purpura in children. J Clin Rheumatol. (2018) 37:1667–73. 10.1007/s10067-017-3864-6 29018973

[17] Grover N Sankhyan N Bisht JP. A five-year review of clinical profile in HSP. J Nepal Med Assoc. (2007) 46:62–5. 18094739

[18] Lei WT Tsai PL Chu SH Kao YH Lin CY Fang LC. Incidence and risk factors for recurrent Henoch-Schonlein purpura in children from a 16-year nationwide database. Pediatr Rheumatol Online J. (2018) 16:25. 10.1186/s12969-018-0247-8 29661187 PMC5902957

[19] Calvo-Río V Hernández JL Ortiz-Sanjuán F Loricera J Palmou-Fontana N González-Vela MC. Relapses in patients with Henoch-Schönlein purpura: Analysis of 417 patients from a single center. Medicine (2016) 95:e4217. 10.1097/MD.0000000000004217 27428226 PMC4956820

[20] Trapani S Micheli A Grisolia F Resti M Chiappini E Falcini F. Henoch Schonlein purpura in childhood: epidemiological and clinical analysis of 150 cases over a 5-year period and review of literature. Semin Arthritis Rheum. (2005) 35:143–53. 10.1016/j.semarthrit.2005.08.007 16325655

[21] Jauhola O Ronkainen J Koskimies O Ala-Houhala M Arikoski P Hölttä T. Clinical course of extrarenal symptoms in Henoch–Schönlein purpura: a 6-month prospective study. Arch Dis Child. (2010) 95:871–6. 10.1136/adc.2009.167874 20371584

[22] Weiss PF Klink AJ Localio R Hall M Hexem K Burnham JM. Corticosteroids may improve clinical outcomes during hospitalization for Henoch-Schönlein purpura. Pediatrics (2010) 126: 674–81. 10.1542/peds.2009-3348 20855386 PMC3518383

[23] Jarasvaraparn C Lertudomphonwanit C Pirojsakul K Worawichawong S Angkathunyakul N Treepongkaruna S. Henoch-Schönlein without Purpura: a case report and review literature. J Med Assoc Thai (2016) 99:441–5. 27396230

[24] Sohagia AB Gunturu SG Tong TR Hertan HI. Henoch-Schonlein Purpura—a case report and review of the literature. Gastroenterology Res Pract. (2010) 2010:597648. 10.1155/2010/597648 20508739 PMC2874920

[25] Possenti I Borali E Longaretti P Bassi LA Cattaneo F Bianchi L. A 10-year-old girl with gastrointestinal hemorrhage. Pediatric Ann. (2015) 44:97–9. 10.3928/00904481-20150313-05 25806725

[26] Karnsakul W Fallon KB Swart S. Exudative hemorrhagic duodenitis as a primary event in a child with Henoch-Schönlein purpura. Clin Gastroenterol Hepatol. (2008) 6:A24. 10.1016/j.cgh.2008.02.019 18407795

[27] Nathan K Gunasekaran TS Berman JH. Recurrent gastrointestinal Henoch-Schönlein purpura. J Clin Gastroenterol. (1999) 29:86–9. 10.1097/00004836-199907000-00022 10405241

